# Heavy Metal Contamination and Its Effects on Ecosystems and Human Health: Challenges and Solutions

**DOI:** 10.3390/toxics13100837

**Published:** 2025-09-30

**Authors:** Daochen Zhu

**Affiliations:** 1 International Joint Laboratory on Synthetic Biology and Biomass Biorefinery, Biofuels Institute, School of the Environment and Safety Engineering, Jiangsu University, Zhenjiang 212013, China; dczhucn@hotmail.com; 2 Jiangsu Collaborative Innovation Center of Technology and Material of Water Treatment, Suzhou University of Science and Technology, Suzhou 215009, China

## 1. Introduction

Heavy metals and other potentially toxic elements (PTEs) constitute a durable class of contaminants because they are non-degradable, widely emitted, and prone to accumulate across environmental compartments [1]. Across the world, the sources of these kinds of contamination are often the same—mining and smelting districts, industrial discharge, traffic-related particulates and road dust, legacy dumps, and belts of intensive agro-industrial activity [2]. This Special Issue aimed to advance an integrated understanding of how such contaminants move across compartments, how speciation and bioavailability govern the risk of contamination, and how remediation and management strategies can be designed to mitigate impacts to the environment and human health. The expansion of urban settlements shrouds this map with a low, chronic haze of inputs, as expanding industrial parks and mixed land uses discharge variable, often metal-bearing, effluents and runoff that conventional WWTPs treat imperfectly without site-specific polishing [3]. Meta analyses converge on the same pattern: mining enriches soils and river sediments; factories feed rivers through persistent wastewater discharge; and cities sustain exposure through dust that recirculates long after the smokestacks quiet down [4,5]. The consequences are familiar, pronounced “hotspot-to-urban” gradients and a slow, steady reloading of downstream sinks even as primary emissions fall. Once contaminants are released, risk is governed less by totals than by speciation and bioavailability, because organisms experience the form that is actually in a solution or at reactive surfaces. Partitioning among dissolved, colloidal, and particulate phases, together with complexation and competition at biological ligands, sets the effective dose. In aquatic systems this principle is operationalized by the Biotic Ligand Model, which underpins U.S. EPA copper criteria and is now evolving beyond equilibrium single-metal fits toward kinetic and mixture-aware formulations [6,7]. For ingestion pathways, in vitro bioaccessibility assays quantify the fraction that becomes soluble under gastrointestinal conditions, with PBET and the SBRC assay widely used in soils and dusts and the Unified BARGE Method formalized as ISO 17924 [8]. Evidence from mining-impacted soils and urban dust shows that reliance on total concentrations can result in risk being poorly estimated, and that pairing operational chemical fractionation with bioaccessibility yields more decision-relevant exposure metrics [9]. Where feasible, linking these measurements to physiologically based pharmacokinetic or toxicokinetic models, such as the IEUBK model for lead, translates an external bioaccessible dose into an internal dose and target organ metrics, tightening uncertainty in risk estimates [10].

The public health burden is substantial and measurable. For lead, no safe exposure level has been identified; the WHO and recent syntheses document enduring neurodevelopmental toxicity, and a 2019 global analysis attributed a loss of about 765 million IQ points in children younger than five to early-life lead exposure [11,12]. Cadmium is a prototypical skeletal toxicant; meta-analyses link exposure to environmental cadmium to lower bone mineral density and higher osteoporosis risk, particularly among postmenopausal women [13,14,15]. Mercury remains on the WHO’s list of ten chemicals of major public health concern, reflecting potent neurotoxicity with heightened vulnerability in the developing fetus. Beyond contemporary emissions, climate-amplified extremes such as floods and wildfires remobilize legacy metal stores in sediments, tailings, and floodplains, producing acute contaminant pulses superimposed on chronic exposure and often intersecting with rapid urban expansion [16]. In several regions, dietary pathways can dominate; cadmium and lead in rice supply chains are recurrent findings, which reinforces the need for mixture-aware, pathway-specific risk management.

Across this landscape, five practical questions keep returning. First, can we separate natural background contaminant levels from human inputs and state the uncertainty clearly? Second, how do we turn total concentration data into quantifications of exposure and dose by bringing in speciation, bioaccessibility, and differences in susceptibility? Third, how can we quantify and predict remobilization at the interface between sediment and water during storms, drying and rewetting, and redox shifts? Fourth, how do we protect food systems, including informal aquaculture and peri-urban agriculture, in places where diet can dominate exposure? Fifth, how do we move from single-chemical point estimates to probabilistic, mixture-aware risk assessments that directly support decisions.

This Special Issue assembles new evidence and tools across these fronts, spanning environmental monitoring and modeling, ecotoxicology and behavior, epidemiology and mechanistic mediation, food chain assessments, and intervention concepts. Together, the contributions provide a compact, methodologically diverse snapshot of where the field stands—and where it needs to go.

## 2. What This Special Issue Adds

River systems and integrated risk indices. One contribution synthesizes PTE contamination across rivers, combining multi-metric ecological indices (e.g., potential ecological risk, a pollutant load index, and a geo-accumulation index) with human health risk resulting from the consumption of aquatic species. Beyond mapping hotspots, the review underscores the need for harmonized metrics and comparable baselines across studies to inform management priorities. Regarding human bone health and endocrine mediation, two epidemiological studies independently link metals to impaired bone mineral density (BMD) and osteoporosis risk, while advancing why these links might arise. A large NHANES analysis reports a negative association between blood manganese and BMD and explores sex steroid hormones (including SHBG and estradiol) as potential mediators, sharpening the mechanistic lens on endocrine disruption and skeletal outcomes. A biomonitoring study in postmenopausal women identifies urinary cadmium and antimony as independent correlates of higher osteoporosis odds, highlighting co-exposures and population vulnerability. Together they illustrate how mixture-aware, mediator-informed epidemiology can move beyond associations and toward plausible pathways. On the subject of organ-specific toxicity and candidate interventions, moving from hazard to mitigation, a nanomedicine study develops a curcumin-loaded nanoemulsion to counter copper-induced neurotoxicity, improving pharmacokinetics and neuroprotection in preclinical models. While such delivery strategies are not a substitute for exposure reduction, they do foreshadow adjunctive, lower-toxicity therapeutics for metal-related disorders.

Biological variability matters, as demonstrated in a zebrafish study, where consistent behavioral phenotypes (bold vs. shy) were found to modulate sensitivity to cadmium and even to a common anthropogenic marker (caffeine), with consequences for locomotion and social behavior. This work reminds us that inter-individual variability, often treated as “noise”, may systematically bias risk estimates if not explicitly designed into experiments and assessments. When it comes to food chain safety in aquatic agroecosystems, seeking data to keep pace with the expansion of nature-based and floating agriculture, a field investigation of lotus cultivated in floating lake gardens quantifies tissue-level accumulation, translocation to edible parts, and health risk indices. The results suggest preferential root accumulation and limited mobility into edible tissues, but also emphasize that chronic exposure and site conditions can shift risk profiles—underscoring the need for crop- and site-specific guidance.

For the remobilization of modeling and background levels, from concentrations to fluxes, a one-dimensional reaction-transport model tracks heavy metal desorption rates and background concentrations in cohesive sediments and reproduces estuarine observations. By coupling dissolved and particulate phases with diffusion, sedimentation, and turbulent exchange, the work offers a transferable boundary module for 3D hydrodynamic models and a pragmatic framework for distinguishing background signals from anthropogenic sources. In terms of deterministic and probabilistic risk, an integrated soil-risk assessment from a karst region finally applies both classical point estimate methods and Monte Carlo simulation, revealing that traditional approaches can underestimate carcinogenic risks (e.g., arsenic and chromium), and demonstrating how target organ toxicity dose metrics refine priority setting. This dual approach aligns assessment practice with the uncertainty and variability inherent in real-world exposures.

## 3. Remaining Gaps and a Forward Program

The next steps are to replace bulk concentrations with dose-relevant measures and to treat exposure as a dynamic mixture that shifts with hydrometeorological extremes. In practice, speciation and partitioning (for dissolved, colloidal, and particulate contaminants, and for complexation and competition) should be measured alongside validated oral and inhalation bioaccessibility to anchor physiologically based toxicokinetic modeling and yield internal dose estimates, rather than proxies. Human studies need to be designed up front for co-exposures (metals with co-occurring organics), effect modification (sex, age, and menopausal status), and mediating biology (hormones and inflammatory markers), with explicit causal frameworks to separate pathways and identify points where intervention alters risk. At the system scale, empirically calibrated desorption and particle–water exchange modules should be embedded within watershed and estuarine models that include hydrology and temperature, enabling event-based sampling, data assimilation, and forecasts of flood- and heat-driven remobilization, in order to clarify the line between background and anthropogenic signals.

Turning evidence into action demands a diversified portfolio and decision tools that acknowledge uncertainty. Source control and treatment, augmented by green infrastructure, remain the first-line defense; in defined subgroups, adjunctive biomedical approaches (for example, chelation when clinically indicated or low-toxicity antioxidant delivery) merit careful evaluation that considers feasibility, adherence, safety, and equity as well as efficacy. Food system safeguards should be built crop- and site-specifically for aquatic and informal agro-ecosystems, combining cultivar selection for low uptake, amendments that immobilize Cd/Pb/As, and risk communication tailored to chronic low-dose exposure. Regulators and communities need transparent, transferable approaches to estimate local background signals with uncertainty bands and to update soil and sediment standards accordingly. Probabilistic practice should become routine, reporting both point estimates and distributions with defensible priors and sensitivity analyses, and should be supported by FAIR data, versioned code, and minimal reporting checklists so that studies are re-analyzable and models portable.

## 4. Closing

The studies collected here advance the field on several fronts, harmonizing risk indices across media, clarifying mechanistic links to health, revealing the role of biological variability, safeguarding food chains, and equipping decision-makers with modern modeling and probabilistic tools. Taken together, they signal a shift from mapping contamination to managing risk: dose-relevant, mixture-aware and climate-attuned science coupled with interventions that regulators and communities can implement. The immediate priorities are standardized metrics and baselines, transparent background estimation, routine probabilistic reporting, and open, reproducible models that translate into measurable reductions in exposure and disease.

## Data Availability

No new data were created or analyzed in this study. Data sharing is not applicable to this article.
